# Early-life hyperoxia-induced Flt3L drives neonatal lung dendritic cell expansion and proinflammatory responses

**DOI:** 10.3389/fimmu.2023.1116675

**Published:** 2023-02-10

**Authors:** Tracy X. Cui, Alexander E. Brady, Ying-Jian Zhang, Christina T. Fulton, Adam M. Goldsmith, Antonia P. Popova

**Affiliations:** Department of Pediatrics, University of Michigan Medical School, Ann Arbor, MI, United States

**Keywords:** prematurity, bronchopulmonary dysplasia (BPD), hyperoxia, dendritic cells, Flt3L

## Abstract

Premature infants with chronic lung disease, bronchopulmonary dysplasia (BPD), develop recurrent cough and wheezing following respiratory viral infections. The mechanisms driving the chronic respiratory symptoms are ill-defined. We have shown that hyperoxic exposure of neonatal mice (a model of BPD) increases the activated lung CD103+ dendritic cells (DCs) and these DCs are required for exaggerated proinflammatory responses to rhinovirus (RV) infection. Since CD103+ DC are essential for specific antiviral responses and their development depends on the growth factor Flt3L, we hypothesized that early-life hyperoxia stimulates Flt3L expression leading to expansion and activation of lung CD103^+^ DCs and this mediates inflammation. We found that hyperoxia numerically increased and induced proinflammatory transcriptional signatures in neonatal lung CD103+ DCs, as well as CD11b^hi^ DCs. Hyperoxia also increased Flt3L expression. Anti-Flt3L antibody blocked CD103+ DC development in normoxic and hyperoxic conditions, and while it did not affect the baseline number of CD11b^hi^ DCs, it neutralized the effect of hyperoxia on these cells. Anti-Flt3L also inhibited hyperoxia-induced proinflammatory responses to RV. In tracheal aspirates from preterm infants mechanically-ventilated for respiratory distress in the first week of life levels of FLT3L, IL-12p40, IL-12p70 and IFN-γ were higher in infants who went on to develop BPD and FLT3L levels positively correlated with proinflammatory cytokines levels. This work highlights the priming effect of early-life hyperoxia on lung DC development and function and the contribution of Flt3L in driving these effects.

## Introduction

Preterm birth survivors with chronic lung disease, bronchopulmonary dysplasia (BPD), have increased risk of recurrent wheezing, airflow obstruction, and cardiopulmonary exercise limitation (1–7). Chronic respiratory symptoms are often triggered by early-life respiratory viral infections, including infection with rhinovirus (RV), which tend to be more severe and lead to hospitalizations or risk of bronchiolitis-associated death (1, 8–12). The precise mechanisms of increased respiratory symptoms and susceptibility to respiratory viral infections are poorly understood (10–13). Higher respiratory morbidity is not associated with atopy (14–16), and cannot be fully explained by the characteristic histopathology of BPD, namely impaired alveolar and vascular development (17, 18). Systemic and airway inflammation, a feature of BPD, points to an immune mechanism (19–23).

Part of the lung innate immune system, dendritic cells (DCs) act as first responders to respiratory viral infections and guide the inflammatory response and viral clearance (24, 25). Lung DCs comprise of three main types: conventional DCs (cDCs) and plasmacytoid DCs are present in steady-state, and monocyte-derived DCs, which are recruited upon inflammation (26–28). The two lung cDC types – CD103+ DCs and CD11b^hi^ DCs – differ based on their phenotypic and functional characteristics (27, 28). Distinguished by CD103 (integrin α_E_) expression, CD103^+^ DCs express Toll-like receptor 3 (TLR3), a receptor for dsRNA, and their development is dependent on the transcription factors Batf3 and interferon regulatory factor 8 (Irf8), whereas CD11b^hi^ DCs express TLR7 and TLR2 and are dependent on Irf4 (29–31). Lung CD103+ DCs are required for anti-viral CD8 T cell responses (24). In addition, during viral infection, lung CD103+ DCs travel to the regional lymph node and present antigens on MHC II to stimulate CD4+ T cell proliferation (24, 25).

The development of lung cDCs, like other nonlymphoid and lymphoid organ cDCs, is dependent on fms-like tyrosine kinase 3 ligand (Flt3L) and the Flt3 receptor (32–34). Both lung CD103+ DCs and CD11b^hi^ DCs are almost completely absent in mice that lack Flt3L (33). Compared to CD11b^hi^ DCs, CD103+ DCs express higher levels of the Flt3 receptor and their development is critically dependent on Flt3L (33). On the other hand, the development of CD11b^hi^ DCs is only partially dependent on the Flt3L/Flt3 axis and depends also on other growth factors and receptor (33). Baseline Flt3L expression in the lung is higher compared to other tissues (35). This is likely required for maintenance of lung cDCs homeostasis (36). Flt3L is induced during lung injury in a mouse model of pulmonary fibrosis (37) or upon infection and results in higher number of DCs (38, 39). Furthermore, systemic administration of Flt3L leads to an increase in lung cDCs and amplifies the inflammatory response to infection (40–43). These findings highlight both the homeostatic and pathogenic roles for Flt3L during lung cDCs development.

The notion that inflammatory conditions induce Flt3L levels and lead to cDC exapansion and activation (38, 39) serves as a basis for our hypothesis that chronic respiratory morbidity in children born premature is due to underlying immune mechanisms. Inflammation precedes the clinical symptoms of BPD (22), and persists during disease progression (44). Exposure to hyperoxia is common for preterm infants, correlates with long-term respiratory symptoms, and induces inflammation (6, 45–48). We have previously shown that hyperoxic exposure of immature mice, a model of BPD, increases the number of lung IL-12-producing CD103^+^ DCs and enhances the inflammatory responses and airway hyperresponsiveness following RV infection (49). We also demonstrated that CD103^+^ DCs are required for neonatal hyperoxia-induced inflammatory responses to RV (50). However, the mechanisms by which early-life hyperoxia modulates CD103^+^ DC development and function are unknown.

We hypothesized that early-life hyperoxia stimulates the expression of the DC growth factor Flt3L leading to expansion and activation of lung CD103^+^ DCs, and this mediates the proinflammatory effects of hyperoxia leading to enhanced responses to RV infection.

## Methods

### Study approval

All mouse experiments were performed following the NIH Guide for the Care and Use of Laboratory Animals recommendations. The animal protocol was approved by the University of Michigan Committee on Use and Care of Animals. The human study was approved by the University of Michigan Institutional Review Board.

### Hyperoxia mouse model and anti-Flt3L antibody treatment

Two-day old wild type C57BL/6J mice (Jackson Laboratories, Bar Harbor, ME) were exposed to air or 75% oxygen for up to 14 days using a polypropylene chamber coupled to an oxygen controller and sensor (BioSpherix, Lacona, NY) (51). Dams were exchanged between air and hyperoxia daily. In selected experiments, mice were *in vivo* treated with an anti-Flt3L antibody (R&D, Minneapolis, MN) or a control IgG (BioXCell, West Lebanon, NH) daily intranasally 0.5mg/kg (in 10ul per mouse) during the exposure to normoxia or hyperoxia. This anti-Flt3L antibody is reported to neutralize the Flt3L-Flt3 signaling *in vitro* and *in vivo* (38, 52, 53). In selected experiments, upon completion of hyperoxic exposure, mice were treated with 30 µl of RV1B (3 x 10^8^ PFU/ml), or sham infection (HeLa cell lysate) administered intranasally. Lungs were analyzed immediately after hyperoxia or 5 days after RV infection.

### Flow cytometry sorting lung CD103+DCs and CD11b+DCs and gene array

Lungs were perfused with PBS containing EDTA (0.5 mM) to remove circulating cells in the vasculature, then minced and digested with Liberase TM (100 µg/mL; Roche, Indianapolis, IN), together with collagenase XI (250 µg/mL), hyaluronidase 1a (1 mg/mL), and DNase I (200 µg/mL; Sigma, St. Louis, MO) for 1 hour at 37°C (54). Cells were filtered and treated with RBC lysis buffer (BD Biosciences, Franklin Lakes NJ) and kept on ice in media containing 10% serum. Dead cells were stained with Pac-Orange Live/Dead fixable dead staining dye (Invitrogen) or DAPI when flow sorting was applied. Lung cells were then stained with fluorescent-labeled antibodies against various leukocyte surface markers (CD45, F4/80, CD11c, CD103, CD11b, CD3ε, CD4, CD8, CD69, and IFN-γ). Appropriate isotype-matched controls and Fluorescence Minus One (FMO) controls were used in all experiments. Antibodies were purchased from Biolegend (San Diego, CA). Cells were fixed and analyzed on a Fortessa (Becton-Dickinson, San Jose, CA) or flow sorted by FACSAria II (BD Biosciences) flow cytometer. Flow sorting purity was >95%. Results were analyzed using FlowJo software (Tree Star, Ashland, OR).

Flow sorted CD103+DCs and CD11b+DCs were used for a gene array (Affymetrix Gene Chip (Affymetrix, CA)) by University of Michigan Sequence Core. RNA was extracted from the sorted cells using the RNeasy Plus Micro Kit (Qiagen, Germantown, MD). The microarray analysis was based on Mouse Gene ST 2.1 strips using the Affy wt-pico kit. The expression values for each gene were calculated using a robust multi-array average (RMA). Weighted linear models were fitted to the data and computed the contrasts of interest. Probesets with variance less than 0.04 were filtered out. Probesets with a fold change of 1.5 or greater and an adjusted p-value of 0.05 or less were selected. *P*-values were adjusted for multiple comparisons using false discovery rate Benjamini and Hochberg. The oligo and limma packages of Bioconductor were impremented in the R statistical environment.

### Quantitative real-time PCR

Mouse whole-lung RNA was prepared using TRIzol (Invitrogen, Carlsbad, CA). Flow sorted cells RNA was extracted using the RNeasy Plus Micro Kit (Qiagen, Germantown, MD). Gene mRNA expression (Flt3, CSF1R, Flt3L, GM-CSF, CD207, SlamF9, Myd88, CXCL16, CXCL9, and IL-12p40) was quantified using SYBR green technology. Primer sequences are available upon request. The level of gene expression was normalized to mRNA of β-actin or GAPDH using the 2^-ΔCT^ algorithm.

For graphic representation, the mRNAs for selected target genes relative to β-actin were plotted multiplied by 10^-3^ as indicated in the figure legends.

### Measurement of cytokines

Whole mouse lung homogenates in PBS with Protease inhibitor cocktail (Roche, Mannheim, Germany) were centrifuged and supernatants analyzed for proinflammatory cytokines. Flt3L and GM-CSF were measured by ELISA (all from R&D Systems, Minneapolis, MN).

### Generation of RV and RV copy number

RV1B (from American Type Culture Collection, Manassas, VA) was grown in HeLa cells, concentrated, partially purified, and titrated as described (55). Viral titers were measured by plaque assay (56). RV copy number was quantified by a Taqman quantitative PCR method with primers and FAM labeled probe that specifically recognizes RV (57). The primer and the probe sequences are available upon request.

### Human tracheal aspirate collection and Multiplex measurement

We examined tracheal aspirates from infants admitted to the C.S. Mott Children’s Hospital Newborn Intensive Care Unit. Entry criteria included gestational age at birth ≤ 32 weeks, mechanical ventilation for respiratory distress, and age ≤ 7 days. Aspirates were collected during routine tracheal suctioning of mechanically ventilated premature infants as described (58). Patient demographics are included in **Table 1**. Human Flt3L, IL-12p40, IL12-p70, IFNr and IL-5 were measured using the tracheal aspirate with a customized Multiplex ELISA (MilliporeSigma, Burlington, MA).

**Table 1:** Patient characteristics.

n	57
Male gender, *n* (% of total)	38 (67)
Gestational age, mean ± SD, wk	27.1 ± 2.2
Birth weight, mean ± SD, g	999 ± 334
Died, *n* (% of total)	1 (2)
BPD or death, *n* (% of total)	36 (63)
BPD, (% of patients evaluated at 36 wk postmenstrual age), n	35 (61)
Day of sampling, median (IQR)	3 ± 2

### Statistical analysis

Data are represented as mean ± SEM or median and interquartile range (IQR). Statistical significance was determined by unpaired *t*-test, nonparametric rank-sum test, one-way ANOVA, as appropriate. Differences were pointed by Newman-Keuls’ multiple comparisons test.

## Results

### Neonatal hyperoxia selectively increases the populations of lung cDCs, CD103+ DCs and CD11b^hi^ DCs

Neonatal hyperoxia impairs alveolar and vascular development (18, 59, 60), however little is known about the effects on neonatal lung immune cell development. We have previously reported that in neonatal mice exposure to hyperoxia for 14 days, a model of BPD, increases the number of lung IL-12-producing CD103+ DCs, and these cells are further increased after RV infection (49). We have also shown that CD103+ DCs are required for hyperoxia-induced proinflammatory responses and airway hyperresponsiveness following RV infection (50). These results implicate hyperoxia-activated neonatal lung CD103+ DCs as a mediator of the inflammatory responses induced by hyperoxia. We have not determined yet the timeline and mechanisms regulating the development of lung CD103+ DCs during early-life hyperoxia.

**Figure 1:**
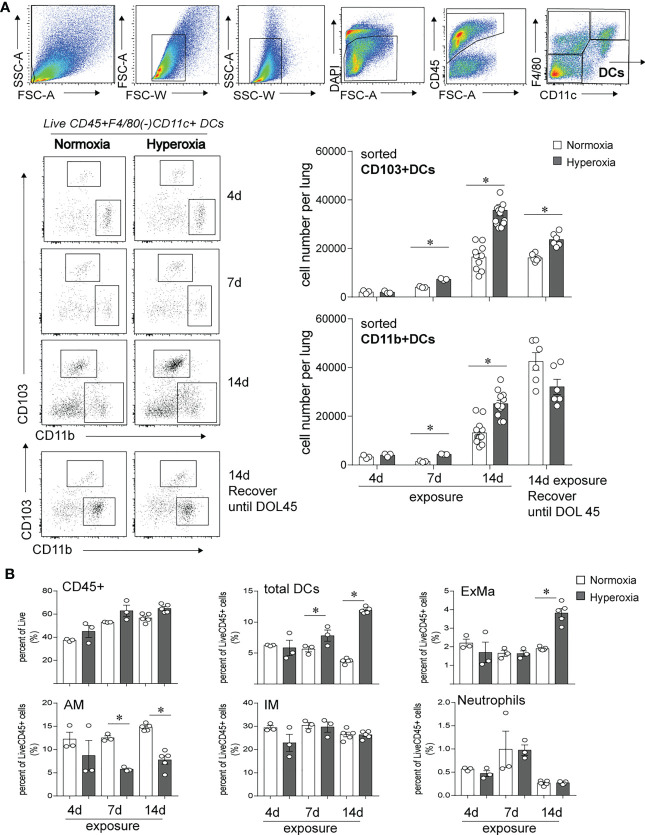
Early-life hyperoxic exposure increased the numbers of CD103+ and CD11b^hi^ lung conventional DCs. **(A)** Two-day-old mice were exposed to normoxia (open bar) or hyperoxia (black bar) for 4, 7 or 14 days (DOL 2 to 16). Lung CD103+ DCs and CD11b^hi^ DCs were determined by flow cytometry sorting methods. Sequential flow gating scheme is presented. Live dendritic cells (DCs) were defined with surface antibodies DAPI-CD45+F4/80-CD11c+ and further differentiated as CD103+ DC or CD11b^hi^ DC. Cell numbers of CD103+ DC and CD11b^hi^ DCs per lung were quantified at 4, 7, 14 days of exposure. In some mice, when hyperoxic exposure (DOL 2 to 16) was discontinued, the mice were recovered in room air until adulthood (DOL 45), then lung CD103+ DCs and CD11b^hi^ DCs were analyzed by the similar flow sorting and quantification. (n = 3-12 per group, *P<0.05, one-way ANOVA). **(B)** CD45+ immune cells, total DCs, Exudative Macrophages (ExMa), Alveolar Macrophages (AM), Interstitial Macrophages (IM), and Neutrophils in lung were also analyzed by flow cytometry at day 4, 7 and 14 of the exposure. These results are representative of two independent experiments. *P<0.05 (one-way ANOVA).

In this study, we investigated the longitudinal effects of neonatal hyperoxia on lung CD103+ DCs and compared with the effects on lung CD11b^hi^ DCs. We exposed two-day old mice to normoxia or hyperoxia (0.75 FiO2) continuously for up to 14 days as described previously (49, 50). We monitored the abundance of lung CD103+DCs and CD11b^hi^ DCs after 4, 7 and 14 days of exposure (**Figure 1**). This period corresponds to rapid alveolar growth in mice (59), and is reminiscent of the exponential increase in alveolar surface area in young human infants (60), thus making this model relevant to understand human BPD pathology. We also examined the long-term effects of neonatal hyperoxia on lung cDC abundance 30 days after hyperoxia was discontinued, on day of life (DOL) 45, corresponding to young adult age. To quantify lung cDCs and critical macrophage populations, we examined lungs by flow cytometry and performed cell sorting of CD103+DCs and CD11b^hi^ DCs to determine the total number of these cell populations per lung (**Figure 1A**). After gating on Live, CD45+ cells, we differentiated lung DCs from alveolar macrophages and interstitial macrophages based on F4/80 and CD11c expression as described previously (49, 61). The fraction of total DCs (CD11c+F4/80- cells) was increased as early as 7 days after hyperoxia began and further increased after 14 days of exposure (**Figure 1B**). Additionally, we examined the two lung cDC subsets, CD103+ DCs and CD11b^hi^ DCs. Under normoxia, the total number of lung CD103+ DCs and CD11b^hi^ DCs increased between exposure day 4 (DOL 6) and exposure day 14 (DOL16) (**Figure 1A**, open bar). In the adult lung (DOL45), the total number of CD103+ DCs remained stable, whereas the total number of CD11b^hi^ DCs further increased. Compared to normoxia, as early as 7 days after hyperoxia the total number of CD103+ DCs and CD11b^hi^ DCs increased significantly, and this number further increased after 14 days of exposure (**Figure 1A**, black bar). Interestingly, the effect of hyperoxia on the number of CD103+DCs, but not CD11b^hi^ DCs persisted in adult lungs 30 days after hyperoxia was discontinued. The effects of hyperoxia on other lung myeloid cells included an increase in the exudative macrophages (F4/80+CD11c+CD11b+, ExMfs) after 14 days exposure and a decrease in alveolar macrophages (F4/80+CD11c+CD11b-, AMs) after 7- and 14-day exposure (**Figure 1B**; **Supplemental Figure 1**). There were no differences in the fraction of interstitial macrophages (IMs) and neutrophils. These results show that neonatal hyperoxia alters the lung innate immune cell milieu with selective expansion of lung CD103+ DCs and CD11b^hi^ DCs.

### Neonatal hyperoxia induces proinflammatory transcriptional signatures in lung CD103+ DCs and CD11b^hi^ DCs

**Figure 2:**
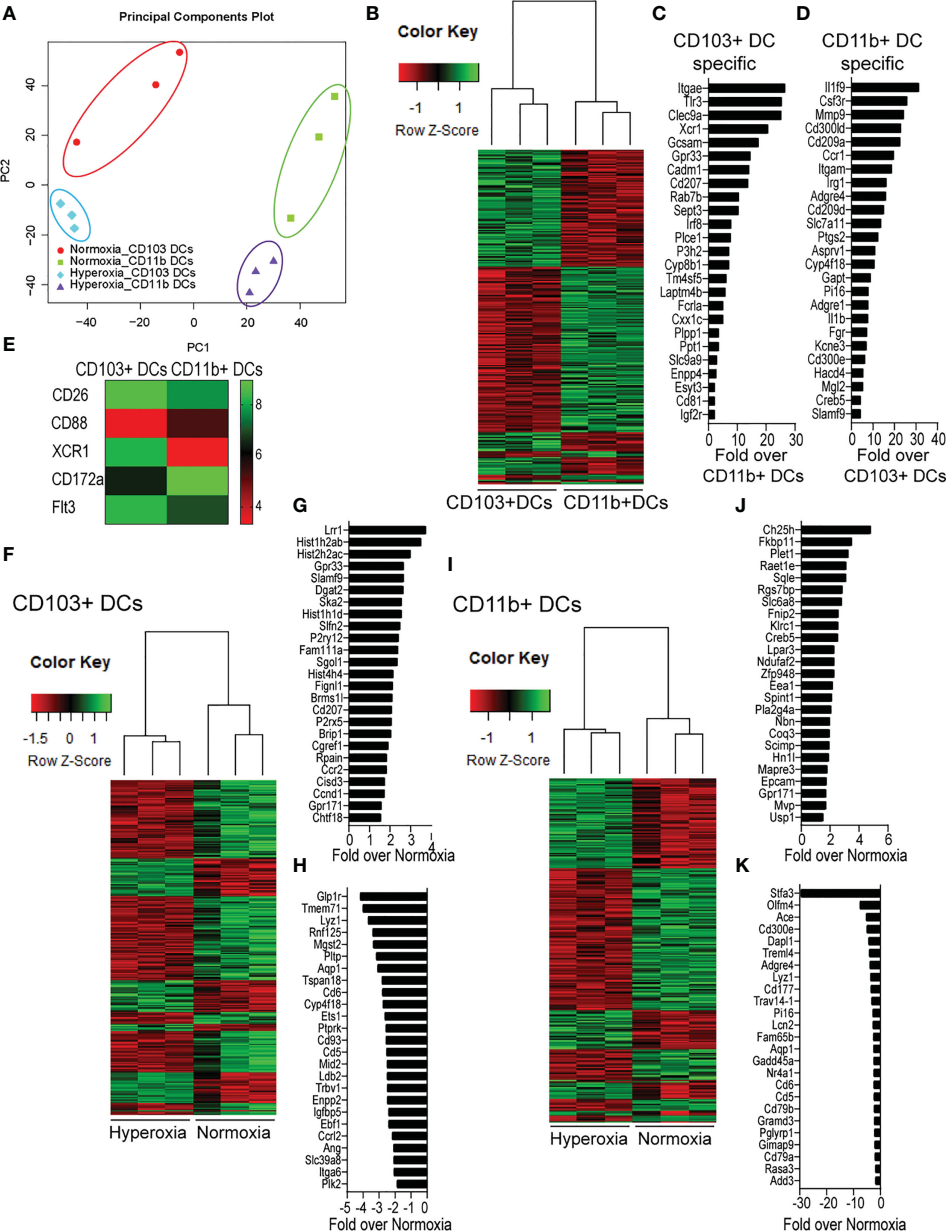
Neonatal hyperoxia alters the gene expression profiles of lung CD103+ DCs and CD11b^hi^ DCs. Flow cytometry sorted CD103+ DCs and CD11b^hi^ DCs from neonatal lungs after 14-days exposure to normoxia or hyperoxia (DOL2 to 16) were subjected to microarray analysis. Each sample contains sorted cells from two separate neonatal mouse lungs. **(A)** Principal component analysis (PCA) on the expression values was used to visualize the overall structure of the data and the first two principal components (PCs) were plotted. Separation on PC1 represents that the largest source of variation is between CD103+ DCs and CD11b^hi^ DCs. Separation on PC2 represents the second largest source of separation is between Normoxia and Hyperoxia condition for each DC subset. **(B)** Heatmap of differentially expressed genes between CD103+ DCs and CD11b^hi^ DCs in normoxic condition. **(C)** Gene signature specific for CD103+ DCs. **(D)** Gene signature specific for CD11b^hi^ DCs. **(E)** Heatmap of expression levels of CD26, CD88, CXR1, CD172a and Flt3. **(F)** Heatmap of differentially expressed genes between Normoxic and Hyperoxic CD103+ DCs. **(G)** Top 25 genes induced by hyperoxia in CD103+DCs. **(H)** Twenty-five most downregulated genes by hyperoxia in CD103+DCs. **(I)** Heatmap of differentially expressed genes between Normoxic and Hyperoxic CD11b^hi^ DCs. **(J)** Top 25 genes induced by hyperoxia in CD11b^hi^ DCs. **(K)** Twenty-five most downregulated genes by hyperoxia in CD11b^hi^ DCs.

To further define the effects of neonatal hyperoxia on lung cDCs, we examined the transcriptional profile of lung CD103+ DCs and CD11b^hi^ DCs isolated from normoxia- and hyperoxia-exposed neonatal lungs. First, we ensured that we can precisely sort the two lung cDC populations from normal neonatal lungs. We established that the differentially expressed genes between neonatal lung CD103+ DCs and CD11b^hi^ DCs closely matched the reported gene signature for lung and other nonlymphoid organ cDC types (30, 62). CD103+ DCs differentially expressed *CD207 (Langerin), CD103 (Itgae), Clec9a, Xcr1, Tlr3, Irf8, Flt3, CD86* and other CD103+ DC-specific genes, while CD11b^hi^ DCs differentially expressed *CD11b (Itgam), CD209d (DC-SIGN), Clec4a1, Tlr7, Tlr2, Cx3cr1, Irf4, CD172a* and other CD11b DC-specific genes (**Figures 2A–E**). Both DC subsets expressed high levels of CD26 and low levels of CD88, consistent with a preDC- and not monocyte-derived origin (**Figure 2E**) (63). Next, we evaluated the effects of hyperoxia on the two lung cDC types. Compared to normoxic lung CD103+ DCs, hyperoxic lung CD103+ DCs differentially expressed 471 genes (**Figure 2F**). We analyzed the differentially expressed genes in hyperoxic lung CD103+ DCs for over-representation of pathways and gene ontology terms using the ConcensusPathDB database (http://cpdb.molgen.mpg.de/) (64, 65). We identified 60 enriched pathway-based sets of genes as defined by the KEGG and Reactome databases (**Supplemental Table S1**). Relevant over-represented pathways reflecting functional changes in the CD103+ DCs after hyperoxia included pathways related to generation of second messenger molecules (8 genes, p-value = 1.85e-09, q-value = 9.54e-07), NF-kappa B signaling pathway (12 genes, p-value = 1.65e-06, q-value = 7.06e-05), signal transduction (64 genes, p-value = 0.000425, q-value = 0.00723), MAPK signaling pathway (14 genes, p-value = 0.00321, q-value = 0.0405) and RAS signaling pathway (12 genes, p-value = 0.00321, q-value = 0.0405) (**Table 2**). We also found 65 enriched gene ontology-based sets of genes (**Supplemental Table S2**). The over-represented sets included gene ontology terms for biological processes related to immune cell activation and development such as leukocyte activation (52 genes, p-value = 1.29e-12, q-value = 4.83e-11), cell motility (68 genes, p-value = 8.76e-12, q-value = 1.46e-10), anatomical structure development (162 genes, p-value = 9.25e-11, q-value = 1.26e-09), response to stress (115 genes, p-value = 4.39e-10, q-value = 4.39e-09) and cytokine production (37 genes, p-value = 1.04e-08, q-value = 9.72e-08) (**Table 3**). Among the top 25 highest upregulated genes were *CD207* (*Langerin*, an endocytic receptor involved in cross-presentation to CD8+ T cells), *Slamf9* (*CD2-F10*, involved in T cell and NK cell activation), the purinergic receptors *P2ry12* and *P2rx5*, oxidative-stress responsive 1 (*Oxsr1*), *Cxcl9, Myd88, Stat2* and others (**Figure 2G**). Among the most downregulated genes were genes involved in cell migration and in vasculature development, including *Itga6, Ang, Aqp1, Igfbp5, Ldb2, Enpp2, Plk2* and others (**Figure 2H**). Comparison of normoxic lung CD11b^hi^ DCs and hyperoxic lung CD11b^hi^ DCs revealed 550 differentially expressed genes (**Figure 2I**). We identified 37 enriched pathway-based sets of genes as defined by the KEGG and Reactome databases (**Supplemental Table S3**). Over-represented pathways reflecting functional changes in the CD11b^hi^ DCs after hyperoxia included pathways related to innate immune system (53 genes, p-value = 4.15e-08, q-value = 4.51e-06), cell surface interactions at the vascular wall (12 genes, p-value = 7.45e-05, q-value = 0.00276) and immunoregulatory interactions between lymphoid and non-lymphoid cells (11 genes, p-value = 7.63e-05, q-value = 0.00276) (**Table 4**). Additionally, we found 55 enriched gene ontology-based sets of genes (**Supplemental Table S4**). The over-represented sets included gene ontology terms for biological processes related to response to stress (143 genes, p-value = 3.26e-12, q-value = 1.61e-10), leukocyte activation (54 genes, p-value = 5.14e-10, q-value = 1.3e-08) and cytokine production (45 genes, p-value = 5.25e-10, q-value = 1.3e-08) (**Table 5**). Among the top 25 most upregulated genes in CD11b^hi^ DCs from hyperoxia-exposed neonatal lungs were predominantly genes involved in regulation of cellular metabolic processes, including *Coq3, Scimp, Mapre3, Usp1, Fnip2, Creb5, Epcam, Pla2g4a, Zfp948, Spint1, Nbn, Mvp* and *Lpar3* (**Figure 2J**). The most downregulated genes in hyperoxic lung CD11b^hi^ DCs included genes regulating immune responses (*Cd177, Ace, Cd6, Cd79a, Gadd45a, Cd79b, Pglyrp1, Treml4*) (**Figure 2K**).

**Table 2:** CD103+ DCs selected overrepresented pathways.

Pathway name	p-value	q-value	Genes
Generation of second messenger molecules	1.85e-09	9.54e-07	Cd3d, Cd3e, Cd4, Lck, Cd247, Cd3g, Lat, Fyb
NF-kappa B signaling pathway	1.65e-06	7.06e-05	Ltb, Ptgs2, Blnk, Cd14, Malt1, Bcl2, Prkcq, Lat, Lbp, Tnfaip3, Lck, Myd88
Signal Transduction	0.000425	0.00723	Tfrc, Dgka, Myd88, Pdgfrb, Tnfaip3, Jak1, Ect2, Insr, Sh2d2a, Bmpr2, Pla2g4a, Spc25, Lpar3, Abca1, Foxo1, Rhoj, Prkch, C5ar1, Tcf7, Plcb4, Tjp1, Hhip, Cenpi, Cav1, Glp1r, Ntrk3, Il2ra, Grb7, Gna14, Lat, F2rl1, Arhgef3, Chn2, Spry1, Gpr4, Rac2, S1pr1, Lef1, Ramp2, Cdh5, Gata3, Arhgap29, Aplnr, Cxcl9, Nedd4l, Sh3kbp1, Gab1, Dusp5, Nr4a1, Calcrl, Rasgrp1, Il2rg, Tek , Ska2, Flt1, P2ry12, Lck, Ednrb, Prkcq, Ccrl2, Sox7, Dock9, Adrb1, Ccnd1
MAPK signaling pathway	0.00321	0.0405	Nr4a1, Dusp5, Pla2g4a, Pdgfrb, Tek, Cd14, Hspa1a, Map3k8, Map4k2, Rac2, Flt1, Insr, Myd88, Rasgrp1
Ras signaling pathway	0.00321	0.0405	Gab1, Pla2g4a, Pdgfrb, Tek, Rapgef5, Ets1, Ets2, Rac2, Lat, Flt1, Insr, Rasgrp1

**Table 3:** CD103+ DCs selected overrepresented gene ontology terms.

Gene ontology term	p-value	q-value	Genes
GO:0045321 leukocyte activation	1.29e-12	4.83e-11	Fcrl1, D6Wsu163e, Icosl, Lrrc32, Igha, Cd2, Il27ra, Malt1, Ager, Bcl2, Cav1, Cd3d, Cd3e, Cd3g, Cd4, Cd5, Cd6, Cd79a, Ccr2, Efnb1, Efnb2, Ephb4, Gata2, Gata3, Nfkbiz, Cd79b, Ighm, Il2ra, Il2rg, Itm2a, Lat, Lbp, Lck, Lef1, Blnk, Myd88, Slc11a1, Prkcq, F2rl1, Rac2, Satb1, Ccl2, Foxp3, Tcf7, Slamf6, Thbs1, Tnfaip3, Tfrc, Tox, Fyb, Rasgrp1, Adgrf5
GO:0048870 cell motility	8.76e-12	1.46e-10	Itga6, Clec14a, Zswim6, Prkd2, P2ry12, Oxsr1, Ace, Ager, Ang, Aqp1, Bcl2, Bmpr2, C5ar1, Cav1, Ccr2, Dapk2, S1pr1, Ednrb, Efnb1, Efnb2, Enpep, Ephb4, Flt1, Gata2, Gata3, Grb7, Nr4a1, Hoxa5, Igfbp5, Insr, Lbp, Ldb2, Lef1, Cxcl9, Myd88, Ntrk3, Nr4a2, Pdgfrb, Enpp2, Pecam1, Prkcq, Pltp, F2rl1, Ptgs2, Ptprg, Ptprk, Rac2, Saa3, Ccl2, Sell, Sema3c, Sema3f, Sorl1, Sox18, Gab1, Ccrl2, Egfl7, Tek, Thbs1, Abhd2, Tie1, Tnfaip3, Mtus1, Ets1, Plk2, Sh3kbp1, Rhoj, Skap1
GO:0048856 anatomical structure development	9.25e-11	1.26e-09	Itga6, Aspn, Calcrl, Clec14a, Zswim6, Pllp, Tmem100, St8sia6, Cecr2, Nrn1, Prkd2, Wdr62, Antxr1, Rasip1, P2ry12, Aff4, Ramp2, Icosl, Scn3b, Spink5, Lrrc32, Mief2, Brip1, Katnb1, Tiparp, Il27ra, Malt1, Ace, Chrnb1, Adrb1, Ager, Ang, Ank3, Aqp1, Bcl2, Bmpr2, C5ar1, Cav1, Ccnd1, Cd3d, Cd3e, Cd3g, Cd4 , Cd79a , Cdh5, Cldn5, Ccr2, Dhfr, Ebf1, Ect2, S1pr1, Ednrb, Efnb1, Efnb2, Emb, Enpep, Epas1, Ephb4, Ablim1, Flt1, Gata2, Gata3, Gpd2, Hhip, Nfkbiz, Nr4a1, Hoxa5, Ndst1, Id3, Cd79b, Igfbp5, Ighm , Il2ra, Il2rg, Insr, Itm2a, Jak1, Lck, Ldb2, Lef1, Ltb, Tm4sf1, Mgp, Cxcl9, Mns1, Meis2, Adam22, Sik1, Myd88, Nfib, Ntrk3, Nr4a2, Nedd4l , Pax5, Pdgfrb, Enpp2, Pecam1, Prkch, Pla2g4a, F2rl1, Ptgis, Ptgs2, Ptprb, Ptprg, Ptprk, Rac2, Satb1, Scd1, Ccl2, Glg1, Sema3c, Sema3f, Foxp3, Slc34a2, Sorl1 , Sox18, Sox7, Gab1, Stat2, Tbx2, Tbx3, Tcf7, Egfl7, Tek, Slamf6, Thbs1, Abhd2, Tie1, Tjp1, Tnfaip3, Tfrc, Dgat2, Tox, Txnip, Foxo1, Ets1, Ets2, Spry1, Gpr4, Gmnn, Plk2, Sh3kbp1, Spint1, Rasgrp1, Rhoj, Abcg2, Fto, Aplnr, Slc7a11, Hpgd, Kdm5b, Adgrf5, Igsf9, Klf7, P2rx5, Klk8, Tapt1, Isg15, Cnn3, Lpar3, 4932438A13Rik, Gpr171
GO:0006950 response to stress	4.39e-10	4.39e-09	Acer2, Calcrl, Ube2t, Cpeb4, Rnf125, Pllp, Ticrr, Mcm10, Neil3, P2ry12, Nfrkb, Spink5, Brip1, Cd209d, Igha, Oxsr1, Il27ra, Malt1, Dapl1, Abca1, Ace, Adrb1, Ager, Ang, Aqp1, Atp1b1, Bcl2, Bmpr2, C5ar1, Cav1, Ccnd1, Cd14, Cd4, Cd6, Cdh5, Ccr2, Dhfr, Rell1, Ect2, Ednrb, Epas1, Flt1, Fmo1, Gata3, Glp1r, Gpr33, Mr1, Nfkbiz, Ndst1, Id3, Igfbp4, Ighm, Chia1, Il2ra, Itih4, Lat, Lbp, Lck, Cd207, Lyz1, Cxcl9, Sik1, Ppp1r15a, Myd88, Slc11a1, Ntrk3, Nr4a2, Nedd4l, Pecam1, Prkcq, Pla2g4a, F2rl1, Cd96, Ptgis, Ptgs2, Ptprk, Saa3, Scd1, Ccl2, Foxp3, Srsf5, Gab1, Stat2, Tbx2, Tbx3, Ccrl2, Tek, Slamf6, Thbd, Thbs1, Abhd2, Tnfaip3, Txnip, Foxo1, Ets1, Mid2, Gpr4, Rpain, Plk2, Rasgrp1, Fto, Map3k8, Map4k2, Slc7a11, Polk, Fignl1, Trim8, P2rx5, Pnma1, Hspa1a, Klk8, Eme2, Isg15, Wfdc1, Pbk
GO:0001816 cytokine production	1.04e-08	9.72e-08	Rnf125, Prkd2, Icosl, Lrrc32, Cd209d, Cd2, Il27ra, Malt1, Abca1, Ager, C5ar1, Cd14, Cd3e, Cd247, Cd4, Cd6, Ccr2, Gata3, Mr1, Chia1, Lbp, Lef1, Ltb, Myd88, Slc11a1, Prkcq, F2rl1, Cd96, Ptgs2, Ccl2, Foxp3, Tek, Slamf6, Thbs1, Tnfaip3, Rasgrp1, Isg15

**Table 4:** CD11b-hi DCs selected overrepresented pathways.

Pathway name	p-value	q-value	Genes
Innate Immune System	4.15e-08	4.51e-06	Olfm4, Sell, Cd4, Aim2, Klrc1, Fabp5, Cyfip2, Pglyrp1, Pglyrp2, Serpinb1a, Slc44a2, Cstb, C5ar1, Itgal, Mvp, Camp, Hp, Slc2a3, Cd247, Ager, Atp6v0a1, Atp6v1c1, Dtx4, Gns, Ltf, Adgre5, Dusp6, Cxcl3, Txnip, Tmc6, Syngr1, Plac8, Cd177, C5ar2, Prkcq, Pecam1, Mmp8, Cd300e, Lcn2, Gzmm, Nod1, Cd19, Cd55, Ptprb, Ear6, Gyg, Txk, Vat1, Lbp, Tmbim1, Mospd2, Cd3g, S100a1
Cell surface interactions at the vascular wall	7.45e-05	0.00276	Slc7a5, Slc7a11, Sell, Thbd, Epcam, Esam, Pecam1, Ighm, Cd177, Itgal, Atp1b1, Itga5
Immunoregulatory interactions between a Lymphoid and a non-Lymphoid cell	0.000425	0.00723	Sell, Treml4, Cd3d, Cd247, Cd300e, Ifitm1, Cd19, Cd3g, Cd200r2, Itgal, Cd3e

**Table 5:** CD11b-hi DCs selected overrepresented gene ontology terms.

Gene ontology term	p-value	q-value	Genes
GO:0006950 response to stress	3.26e-12	1.61e-10	Plet1, Fgd4, Pde2a, Calcrl, Oxct1, Psmc6, Rnaseh2b, Dusp6, Rnf125, Chd6, Myof, Gadd45a, Ifitm1, Cd177, Cxcl16, Vps33b, Traf3ip2, Kynu, Optn, Pnpt1, Usp1, Ubash3a, Setx, Trip10, F13a1, Ffar4, Igha , Dst , Cd200r2, Nod1, Cd200r4, Dapl1, Abca1, Ace, Ager, Ang, Apoe, Aqp1, Atp1b1, Fxyd2, Slc7a2, Fnip2, C5ar1, Cd37, Cd38, Cd4, Cd6, Prdx1, Cdh5, Cldn1, Plac8, Camp, Cd55 , Zc3hav1, F2rl2, Fmo1, Fosl1, Gch1, Gja4, Gclm, Glp1r, Cxcl3, Pdpn, Gsr, Gstm5, Smurf1, Foxf1, Hp, Postn, Ndst1, Igf1r, Igfbp4, Ighm, Il1a, Ifitm6, Itga5, Supt16, Mdfic, Lbp, Lcn2, Lipa, Lmnb1, Loxl3, Xcl1, Ltf, Lyz1, Marco, Mgmt, Cxcl9, Mmp8, Tmf1, Cd200, Sik1, Naip2, Nlrp12, Ngp, Pecam1, Pik3cd, Prkcq, Pla2g4a, F2rl1, Pglyrp2, Bok, Rab20, Rad51, Rasa3 , Rb1, Rfc1, Saa3, Ccl17, Ccl9, Sel1l, Foxp3, Srsf5, Spp1, Stxbp3, Tbx3, Thbd, Thbs1, Cd27, Pglyrp1, Txk, Vegfc, Zmat3, Aim2, Txnip, Ets1, Hilpda, C5ar2, Trib1, Map4k2, Slc7a11, Asns, Nbn, Cd163, Treml4, Hexim1, Map4k3, P2rx5, Klk8, Raet1e, Isg15, Dusp10
GO:0045321 leukocyte activation	5.14e-10	1.30e-08	Cd177, Mzb1, Icosl, Vsir, Igha, Ms4a1, Ager, Slc7a2, Cd19, Cd37, Cd38, Cd3d, Cd3e, Cd3g, Cd4, Cd5, Cd6, Cd79a, Prdx1, Bank1, Foxf1, Cd79b, Ighm, Itgal, Itm2a, Lbp, Lef1, Loxl3, Xcl1, Ly6d, Mmp8, Cd200, Pik3cd, Prkcq, F2rl1, Pglyrp2, Satb1, Foxp3, Stxbp3, Tcf7, Bcl11b, Thbs1, Cd27, Pglyrp1, Tpd52, Txk, Ikzf1, Ikzf3, Fyb, Nbn, Cd274, Adgrf5, Raet1e, Dusp10
GO:0001816 cytokine production	5.25e-10	1.30e-08	Rnf128, Rnf125, Prkd2, Icosl, Optn, Ubash3a, Vsir, Ffar4, Nod1, Abca1, Ager, Anxa4, C5ar1, Cd3e, Cd247, Cd4, Cd6, Bank1, Zc3hav1, Postn, Il1a, Lbp, Lef1, Lipa, Xcl1, Ltb, Ltf, Mmp8, Tmf1, Cd200, Nlrp12, Prkcq, F2rl1, Pglyrp2, Foxp3, Trib2, Thbs1, Cd27, Pglyrp1, Txk , Aim2, Hilpda, C5ar2, Cd274, Isg15

**Figure 3:**
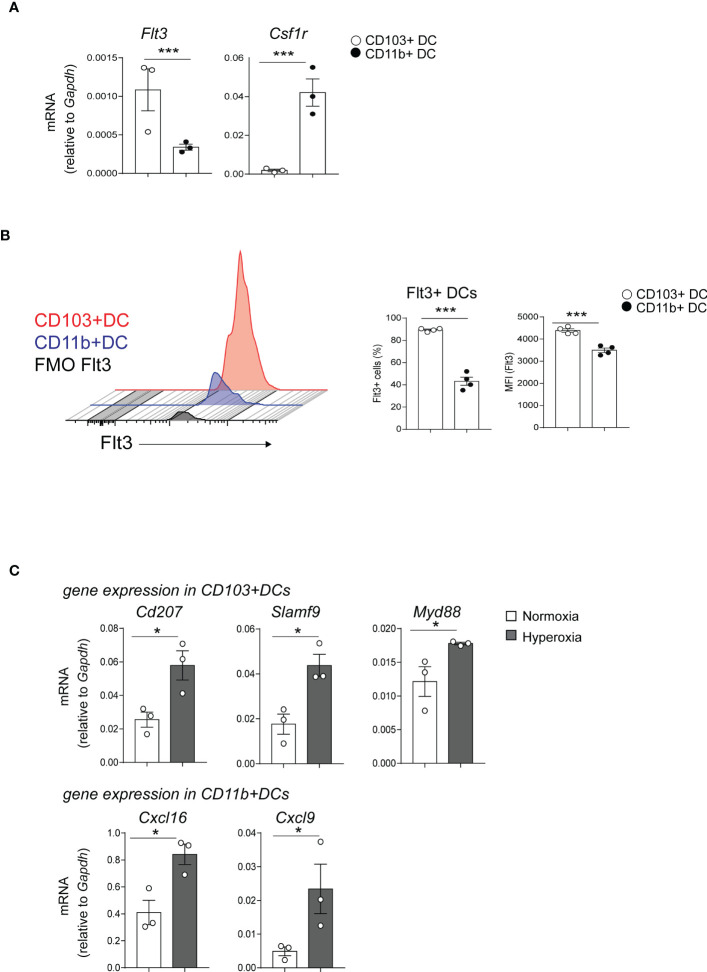
Differentially expressed genes in CD103+ DCs and CD11b^hi^ DCs determine their development and immune function. **(A)** Flt3 and Csf1r mRNA expression was quantified by qPCR in CD103+ DCs (white circle) or CD11b^hi^ DCs (black circle), flow sorted from normoxia-exposed neonatal lungs (DOL16). Each sample contains sorted cells from two separate neonatal mouse lungs. Unpaired *t*-test was performed to test the significance. *** indicates p<0.001. **(B)** Histograms demonstrate Flt3 protein expression in CD103+ DCs (red) and CD11b+ DCs (blue). Unstained control (defined by FMO) is presented in gray. The fraction of Flt3+ CD103+ DCs (open circle) or CD11b^hi^ DCs (black circle) in neonatal lung (DOL 9) was defined by flow cytometry analysis. Mean Florescence Intensity (MFI) of Flt3 was also quantified. These results are representative of two independent experiments. Unpaired *t*-test was performed to test the significance. *** indicates the significant level p<0.001. **(C)**. CD103+ DCs and CD11b^hi^ DCs were flow sorted from neonatal lungs after 14-day exposure to normaxia (open bar) or hyperoxia (black bar). Selected differentially expressed genes in CD103+ DCs (CD207, SlamF9 and Myd88) and in CD11b^hi^ DCs (CXCL16 and CXCL9) were quantified with qPCR. Each sample contains sorted cells from two separate neonatal mouse lungs. Total number of six neonatal mouse lungs per group were studied. *P<0.05 (unpaired *t*-test).

Next, we sought to confirm the microarray results. First, we focused on examining the expression of the two receptors that regulate cDCs development, Flt3 and CSF1R in normal conditions (33). Flt3 was among the differentially expressed genes between neonatal lung CD103+ DCs and CD11b^hi^ DCs (**Figures 2B, E**). Using qPCR, we confirmed that Flt3 mRNA expression in flow sorted neonatal lung CD103+ DCs was significantly higher compared to flow sorted CD11b^hi^ DCs. (**Figure 3A**). As previously shown in adult CD11b^hi^ DCs (33), neonatal lung CD11b^hi^ DCs expressed higher levels of *Csf1r* (**Figure 3A**). Flow cytometry analysis of lung cDCs at DOL 9 confirmed that most CD103+ DCs expressed Flt3 protein compared to less than 50% of the CD11b^hi^ DCs (**Figure 3B**). The average Flt3 protein expression per cell presented as mean fluorescence intensity (MFI) was also higher in CD103+ DCs compared to CD11b^hi^ DCs (**Figure 3B**).

Additionally, the microarray showed that hyperoxia induced the expression of proinflammatory genes in CD103+ DCs and CD11b^hi^ DCs. Using qPCR, we confirmed that hyperoxic CD103+ DCs expressed higher levels of *CD207, Slamf9* and *Myd88* mRNA compared to normoxic CD103+ DCs. As for the CD11b^hi^ DCs, hyperoxia induced the mRNA expression of *Cxcl9* and *Cxcl16* (**Figure 3C**).

Together these results indicate that early-life hyperoxia not only numerically increases lung cDCs, but also induces unique proinflammatory transcriptional signatures in each cDC subset. These results also show higher Flt3 expression in CD103+ DCs, which may confer a developmental advantage upon stimulation with Flt3 ligand.

### Early-life exposure to hyperoxia induces lung Flt3L expression

**Figure 4:**
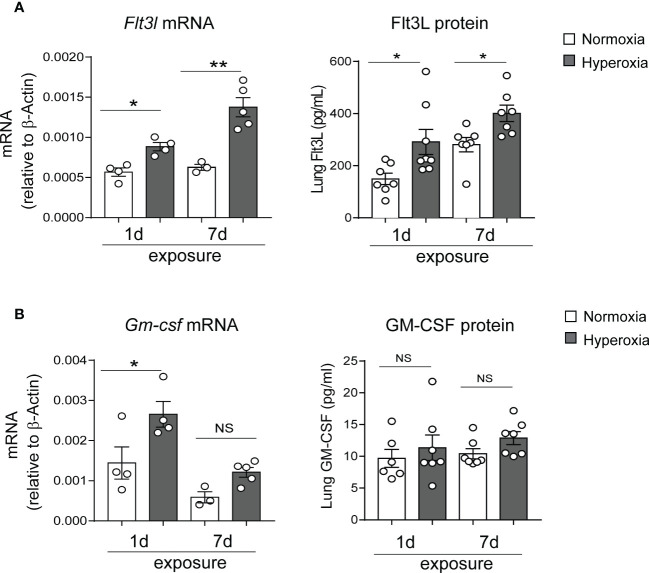
Hyperoxic exposure increased Flt3L but not GM-CSF levels in neonatal mouse lung. Whole lung tissue was homogenized in PBS after 1d and 7d of normoxia (white bar) or hyperoxia (black bar) exposure. Proteins were measured by ELISA in the supernatant. RNA was extracted from part of the homogenate and was used for qPCR. Flt3L **(A)** and GM-CSF **(B)** protein levels and mRNA expression were quantified. These results are representative of two independent experiments. *P<0.05 or **P<0.01 (one-way ANOVA), NS, non-significant.

To investigate the mechanisms by which early-life hyperoxia induces lung cDC expansion and activation, we quantified the expression of the DC growth factors Flt3L and granulocyte-macrophage colony-stimulating factor (GM-CSF) in normoxia- and hyperoxia-exposed neonatal lungs. Flt3L plays a critical role in CD103+ DC development and partially regulates the development of CD11b^hi^ DCs (33). GM-CSF directs differentiation of DCs *in vitro* (66), and regulates nonlymphoid tissue DC development (67). Additionally, GM-CSF is a key driver of chronic lung inflammation contributing to asthma development (68). We found that exposure of two-day old mice to hyperoxia for 1 and 7 days significantly increased Flt3L mRNA and protein levels (**Figure 4A**), whereas the expression pattern of GM-CSF was different. GM-CSF mRNA expression was significantly induced after 1 day but not after 7 days of hyperoxia and GM-CSF protein levels were not affected by hyperoxia (**Figure 4B**). These results indicate that early-life hyperoxia selectively induces the Flt3L/Flt3 pathway.

### Neutralization of Flt3L *in vivo* blocks lung CD103+ DC development in normoxic and hyperoxic conditions and attenuates hyperoxia-induced IL-12p40 expression in the neonatal lung

**Figure 5:**
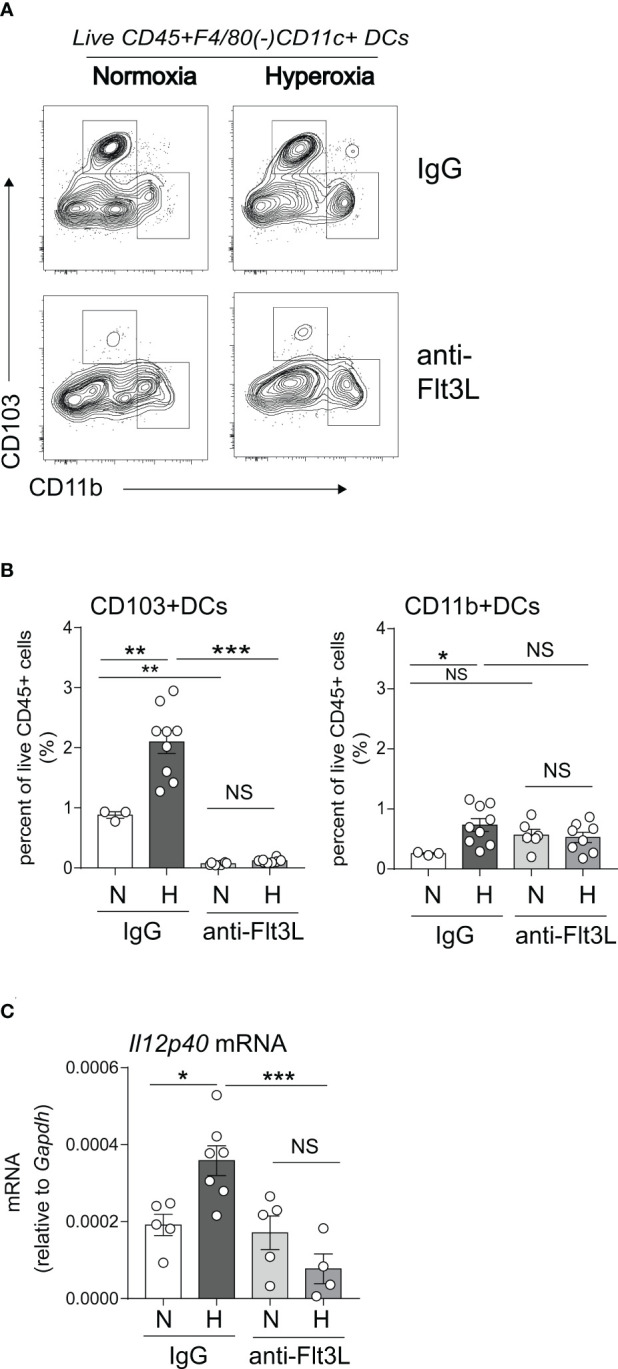
*In vivo* blockade of Flt3L with anti-Flt3L reduces the number of CD103+ DCs in neonatal lung and attenuates the hyperoxia-induced IL-12p40 expression. **(A, B)**. Two-day-old mice were exposed to normoxia or hyperoxia and treated with IgG or anti-Flt3L once daily. After 11 days of hyperoxia exposure and daily anti-Flt3L treatment, total lung CD103+ DCs and CD11b^hi^ DCs were determined and quantified by flow cytometry. Anti-Flt3L not only blocked hyperoxia induced CD103+ DCs numbers in lung but also blocked CD103+ DCs in nomoxic condition. On the other hand, Anti-Flt3L only blocked hyperoxia induced CD11b^hi^ DCs in lung. **(C)**. In some experiments, IL12p40 mRNA expression was measured in whole lung homogenates. Anti-Flt3L blocked hyperoxia-induced IL12p40 mRNA expression in neonatal lung. These results are representative of two independent experiments. Significant levels were indicated by *P<0.05, **P<0.01, ***P<0.001 (one-way ANOVA), NS, non-significant.

We sought to determine if Flt3L is required for neonatal lung cDC development under normoxic and hyperoxic conditions. Neonatal mice were exposed to normoxia or hyperoxia from DOL2 to DOL11 and treated daily with anti-Flt3L or isotype control IgG. On DOL14, lung CD103+ DCs and CD11b^hi^ DCs were quantified by flow cytometry (**Figures 5A, B**). Total lung DCs were identified by sequential gating of live, CD45+, F4/80-, CD11c+ cells. The two types of lung cDCs were identified based on CD103 and CD11b expression. Like the results shown in **Figure 1**, in IgG-treated mice hyperoxia increased the fraction of CD103+ DCs and CD11b^hi^ DCs. Anti-Flt3L treatment significantly reduced the lung CD103+DCs in normoxic and hyperoxic conditions (**Figures 5A, B**). Anti-Flt3L did not affect the fraction of CD11b^hi^ DCs in normoxic neonatal lungs but attenuated the effect of hyperoxia on these cells. These results are consistent with the notion that Flt3L is critically required for neonatal lung CD103+ DC development and for the effects of hyperoxia on this cell population.

We have previously shown that neonatal hyperoxia induces the expression of the proinflammatory IL-12p40 cytokine in whole lung and this increase is dependent on Batf3, a transcription factor required for CD103+ DC development (49, 50). We examined the effects of Flt3L neutralization on whole lung IL-12p40 expression under normoxic and hyperoxic conditions. We found that anti-Flt3L treatment blocked hyperoxia-induced IL-12p40 mRNA expression (**Figure 5C**). These results indicate that Flt3L-dependent DCs are required for hyperoxia-induced proinflammatory IL-12p40 expression.

### In hyperoxia-exposed neonatal mice, *in vivo* blockade of Flt3L attenuates CD4+ and CD8+ T cell activation following RV infection

**Figure 6:**
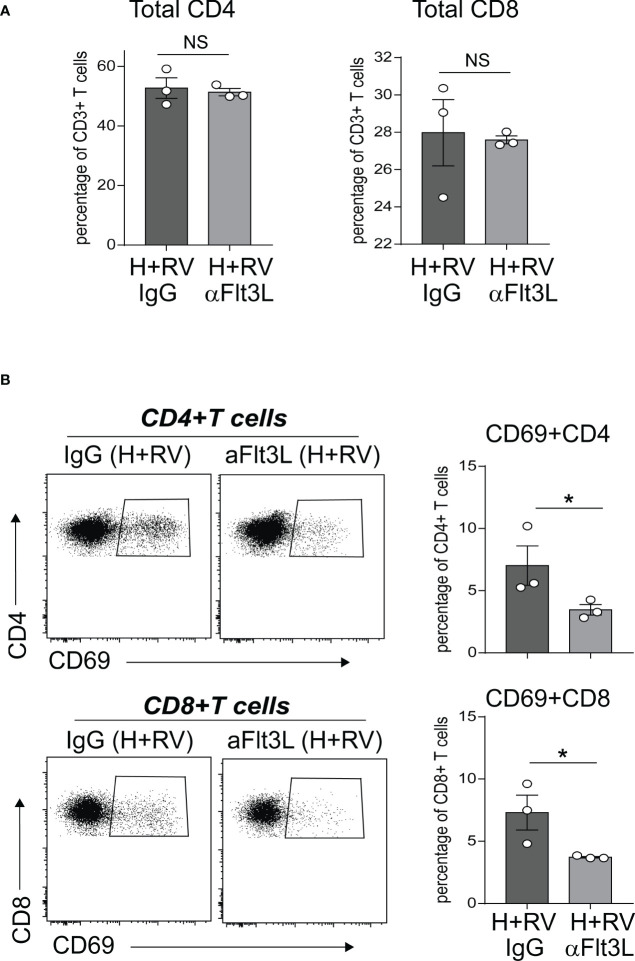
*In vivo* blockade of Flt3L with anti-Flt3L attenuates hyperoxia-induced inflammatory response to RV. Two-day-old mice were exposed to hyperoxia and treated with anti-Flt3L or IgG once daily. After 11 days of hyperoxia exposure and antiFlt3L treatment, mice were inoculated with RV intranasally. Five days later, total and activated CD69+ T cells in lungs were analyzed by flow cytometry. Total CD4 and CD8 T cells **(A)**, as well as CD69+CD4+ and CD69+CD8+ T cells **(B)** were quantified in lung. Each dot is representative of two immature mouse lungs. *P<0.05 (one-way ANOVA), NS, non-significant.

In extensive prior studies, we have reported that neonatal hyperoxia induces proinflammatory lung CD4+ T cell activation following RV infection (49), and that CD103+ DCs are required for these effects (50). Above we showed that anti-Flt3L treatment significantly reduced the number of lung CD103+DCs in normoxic and hyperoxic neonatal mice. We sought to evaluate the effect of *in vivo* blockade of Flt3L on lung T cell activation in hyperoxia-exposed, RV-infected neonatal mice. We exposed neonatal wild-type mice to hyperoxia from DOL 2 to 11 and treated them with anti-Flt3L or isotype control IgG daily. Upon discontinuing the exposure we inoculated the mice with RV (RV1B, 3X10^7^ PFU) or sham, administered intranasally. Using flow cytometry, we examined lungs five days after RV infection and quantified the frequencies of CD4+ T cells and CD8+ T cells expressing CD69, T cell activation marker. Lung T cells were identified by sequential gating for cells that are Live CD45+ CD3ε+ and either CD4+ or CD8+ (**Supplemental Figure 2**). Anti-Flt3L treatment did not affect the frequencies of total CD4+ T cells and CD8+ T cells (**Figure 6A**). Compared with IgG-treated mice, anti-Flt3L-treated neonatal mice had significantly lower frequency of activated CD69+ CD4+ and CD8+ T cells (**Figure 6B**). There was no difference in RV copy numbers between IgG and anti-Flt3L treatment groups (**Supplemental Figure 3**). The results indicate that the effect of hyperoxia on lung CD4+ and CD8+ T cell activation following RV infection is mediated by Flt3L-expanded/activated CD103+DCs.

### Levels of FLT3L and proinflammatory cytokines IL-12p40, IL-12p70 and IFN-γ in preterm infant airways

**Figure 7:**
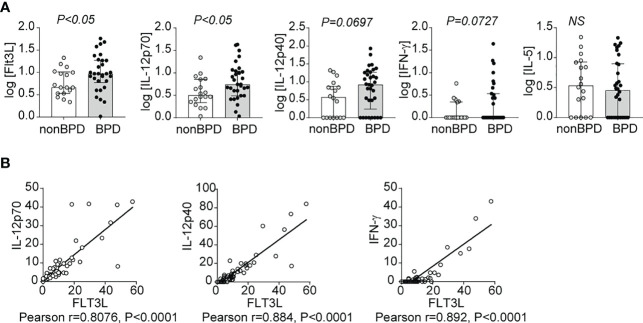
Inflammatory cytokines IL-12p40, IL-12p70 and IFN-γ as well as Flt3L are increased in tracheal aspirates of premature infants with respiratory distress who go on to develop BPD. Mechanically-ventilated premature infants with respiratory distress were recruited to the study. Tracheal aspirates were collected during the first week of life. Multiplex ELISA was performed to measure proinflammatory cytokine IL-12p40, IL-12p70, IFN-γ and IL-5 along with Flt3L protein levels. The cytokine data were log transformed to reduce skewness. BPD was diagnosed based on the requirement for supplemental oxygen or positive pressure ventilation at 36 weeks postmenstrual age. Each protein was compared between infants who went on to develop BPD and those who did not **(A)**. *P<0.05, unpaired *t*-test, NS, non-significant. The association between Flt3L with IL-12p70, IL-12p40, or IFN-γ levels was determined by Pearson’s correlation analysis **(B)**.

To establish the relevance of our findings to human disease, we measured levels of FLT3L, IL-12p70, IL-12p40 and IFN-γ in tracheal aspirates from human preterm infants mechanically-ventilated for respiratory distress in the first week of life. Patient characteristics are described in **Table 1**. We defined BPD based on the need for supplemental oxygen at 36 weeks postmenstrual age. For prematurely born infants, death before 36 weeks postmenstrual age is a competing outcome for BPD and may confound a cause-specific analysis, therefore we combined both outcomes. We found that tracheal aspirates from premature infants who go on to develop BPD have significantly higher FLT3L and IL-12p70 protein levels compared to aspirates from infants who do not develop BPD (**Figure 7A**). IL-12p40 and IFN-γ protein levels also showed a similar trend with borderline statistical significance, whereas levels of IL-5, a type 2 cytokine, were similar (**Figure 7A**). Additionally, FLT3L protein levels positively correlated with levels of IL-12p70, IL-12p40 and IFN-γ (**Figure 7B**). These results suggest that higher levels of FLT3L and proinflammatory cytokines IL-12p70, IL-12p40 and IFN-γ are associated with BPD diagnosis, a predictor of higher chronic respiratory morbidity.

## Discussion

In this study we investigated the effects of early-life hyperoxia on lung cDC development and transcriptional profiles, and the patterns of DC growth factor expression in an experimental model of BPD. We also assessed the capacity of Flt3L neutralizing antibody to modulate the effects of early-life hyperoxia on lung cDCs and proinflammatory responses. We present novel findings that prolonged exposure to hyperoxia in early life increases the populations of lung CD103+ DCs and CD11b^hi^ DCs, and unlike CD11b^hi^ DCs, CD103+ DCs remain increased in adult age, DOL 45 in mice, indicating long lasting effects of hyperoxia. We also show that neonatal hyperoxia induces proinflammatory transcriptional signatures in both CD103+ DCs and CD11b^hi^ DCs. Our results further demonstrate that early-life hyperoxia induces lung Flt3L expression, whereas the expression of GM-CSF is unchanged. Moreover, we establish that anti-Flt3L treatment during hyperoxia 1) blocks the development of lung CD103+ DCs in normoxic and hyperoxic conditions, and while it does not affect CD11b^hi^ DCs at baseline, it neutralizes the effect of hyperoxia on these cells, 2) inhibits hyperoxia-induced IL-12p40 mRNA expression in the neonatal lung, and 3) prevents CD4+ T cell and CD8+ T cell activation in hyperoxia-exposed mice infected with RV. Taken together, these results indicate that Flt3L is a primary mediator of early-life hyperoxia-induced lung cDC expansion and activation, proinflammatory cytokine expression, and impaired alveolar growth. Finally, we show that in tracheal aspirates from human preterm infants mechanically-ventilated for respiratory distress in the first week of life, levels of FLT3L, IL-12p40, IL-12p70 and IFN-γ are higher in infants who go on to develop BPD compared to infants who do not develop BPD. Also, tracheal aspirate FLT3L levels positively correlate with the levels of the proinflammatory cytokines IL-12p40, IL-12p70 and IFN-γ.

Early-life cumulative oxygen exposure and elevated proinflammatory cytokine levels in preterm infants (21, 22, 45, 69) are associated with development of long-term respiratory symptoms and increased susceptibility to respiratory viral infections (10–12). These exposures occur during a period of rapid alveolar growth and contribute to the histopathological changes observed in the “new”, post-surfactant BPD, including hypoalveolarization, dysmorphic vasculature, and absence of airway structural changes (18, 60). However, the effect of hyperoxia on the development of the neonatal lung innate immune system and in particular lung cDCs, the main antigen presenting cells responsible for antiviral responses, is poorly understood.

In this study, we demonstrate that cDCs populate the lungs and increase in number during the first two weeks of life in mice. This occurs during rapid alveolar growth (70), and suggests that lung cDC development is a key feature of early postnatal lung development. We show that hyperoxia accelerates the increase in CD103+ DCs and CD11b^hi^ DCs. At the same time hyperoxia leads to hypoalveolarization. Thus, either lung cDC development is independent of alveolarization or hyperoxia induces changes in lung cDC phenotype that impair alveolar growth. We show that both CD103+ DCs and CD11b^hi^ DCs demonstrate unique proinflammatory transcriptional profiles after hyperoxia. Differentially expressed genes in CD103+ DCs and CD11b^hi^ DCs also include genes involved in development. Together these data support the notion that not the increased number per se but an altered lung cDC phenotype is responsible for the proinflammatory effects of hyperoxia and contributes to impaired lung development. Our findings that both cDC subsets in the neonatal lung acquire proinflammatory phenotype upon exposure to hyperoxia contrast with previous findings in mouse model of fibrosis that demonstrate elevated Flt3L increases lung CD11b^hi^ DC mobilisation to limit the degree of lung fibrosis by secreting repair factors (37). These differences may reflect age- or mechanism-specific lung cDC responses. In this manuscript, we also show that higher number of CD103+ DCs, but not CD11b^hi^ DCs, persists in adult lungs, weeks after hyperoxia is discontinued. It remains to be determined if the long-lasting increase in lung CD103+ DCs is due to accelerated recruitment or local expansion and if this contributes to sustained inflammation.

The development of mature lung cDCs is dependent on the Flt3L-Flt3 signaling pathway (33). Fate-mapping experiments have shown that almost all lung CD103+ DCs and about half of lung CD11b^hi^ DCs are derived from common DC progenitors that express the tyrosine kinase receptor Flt3 (71, 72). Beyond the lung, similar mechanisms govern cDC development in other nonlymphoid organs such as skin and gut, as well as in lymphoid organs (32, 33, 73). Flt3L stimulation *in vivo* induces cDC expansion (40, 74, 75). Additionally, the role of Flt3L in DC homeostasis is supported by studies showing that treatment of bone marrow progenitors with Flt3L *in vitro* also leads to development of mature DCs (71, 74, 76–78). Moreover, the essential role of FLT3L in human cDC differentiation has recently been demonstrated *in vitro* using human cord blood (79, 80), and *in vivo* using humanized mice (81). Other factors that control DC development are GM-CSF and macrophage colony-stimulating factor (M-CSF). In homeostatic conditions, mice with absent GM-CSF or GM-CSF receptor have significantly reduced lung CD103+ DCs, but not CD11b^hi^ DCs (67). We found that Flt3L protein levels in normal neonatal mice increased between day 1 and 7. Hyperoxia further increased Flt3L mRNA and protein levels at these time points. We did not observe similar patterns in GM-CSF protein expression both during normal development and during hyperoxia. These results further reaffirm that Flt3L, but not GM-CSF, is a primary regulator of neonatal lung cDC development and a key mediator of the effects of hyperoxia on these DCs.

Our results demonstrate that Flt3 receptor expression is higher on CD103+DCs compared to CD11b^hi^ DCs. Thus, CD103+ DCs are likely more sensitive to the hyperoxia-induced Flt3L expression levels. It is also possible that the lower Flt3 expression on CD11b^hi^ DCs renders higher sensitivity to other DC growth factors, analogous to the difference observed between Flt3 null and Flt3L null mice (82). This notion is supported by our current observation that the expression of an alternative DC growth factor receptor, CSF1R, is significantly higher in CD11b^hi^ DCs compared to CD103+ DCs.

Since the Flt3L-Flt3 signaling pathway is critical for lung CD103+ DC development (33), we assessed the effect of blocking Flt3L on neonatal lung responses to hyperoxia. The requirement of Flt3L to promote human progenitor cell expansion using specific neutralizing antibody has been demonstrated *in vitro* (52, 53). Using Flt3L neutralizing antibody, we demonstrate a unique requirement for hyperoxia-induced Flt3L to induce lung cDCs expansion and activation, and promote proinflammatory responses. These results further extend our previous findings that CD103+ DCs are required for hyperoxia-induced inflammation (50).

In this study, we identified CD103+ DCs as CD103 high, CD11b low DCs (49, 50, 61). Recent reports describe two phenotypically and functionally distinct lung migratory CD103+ DC populations, CD103lo and CD103hi DCs, following neonatal respiratory viral infection (83, 84). Both populations are absent in Batf3-/- mice and unlike CD103hi DCs, CD103lo DCs express lower levels of lineage and maturation markers, including costimulatory molecules, suggesting they are phenotypically immature and functionally limited (84). While we focus on mature CD103hi DCs, we recognize that our analysis may have underestimated the effects of hyperoxia on other less mature DCs, including CD103lo DCs. Future research is required to determine the effect of neonatal hyperoxia on lung cDC progenitors and their precursors in the bone marrow.

In this report, we determined that Flt3L is required for the effects of hyperoxia on lung CD103+ DC and CD11b^hi^ expansion and activation, however we did not define the mechanisms driving increased Flt3L expression in the lungs during early-life hyperoxia. We acknowledge that defining the processes responsible for hyperoxia-induced Flt3L expression would yield an opportunity to inhibit this phenomenon while preserving normal cDC development. This alternative is especially attractive as both CD103+ DCs and CD11b^hi^ DCs are critical mediators of CD8+ and CD4+ T cell responses during viral infections (24, 29, 85, 86). Nevertheless, the capacity of hyperoxia to induce expression of the DC growth factor Flt3L and accelerate the developmental increase and proinflammatory activation of lung DCs contributing inflammatory responses to RV has not been, to our knowledge, previously recognized.

Collectively, our results demonstrate an essential role for early-life hyperoxia-induced Flt3L in neonatal lung cDC development and responses to promote inflammation. Our findings in human preterm infants with respiratory distress show higher levels of Flt3L and other proinflammatory cytokines in tracheal aspirates from infants who go on to develop BPD. These findings provide a basis to focus future research on targeting specific inflammatory mediators to prevent or treat BPD.

## Data availability statement

The data presented in the study are deposited in the Gene Expression Omnibus repository, accession number GSE223701.

## Ethics statement

The studies involving human participants were reviewed and approved by University of Michigan Institutional Review Board. Written informed consent to participate in this study was provided by the participants’ legal guardian/next of kin. The animal study was reviewed and approved by University of Michigan Institutional Animal Care & Use Committee.

## Author contributions

TC and AP designed research and experiments. TC, AB, Y-JZ, CF and AG performed experiments. TC and AP analyzed experiments. TC and AP wrote the manuscript. All authors have read and approved the manuscript. All authors contributed to the article and approved the submitted version.
