# Supplementary figures and images for: Early-life hyperoxia-induced Flt3L drives neonatal lung dendritic cell expansion and proinflammatory responses
Source: Front Immunol. 2023 Feb 10;14:1116675. doi: 10.3389/fimmu.2023.1116675 (PMC9950736; doi:10.3389/fimmu.2023.1116675)

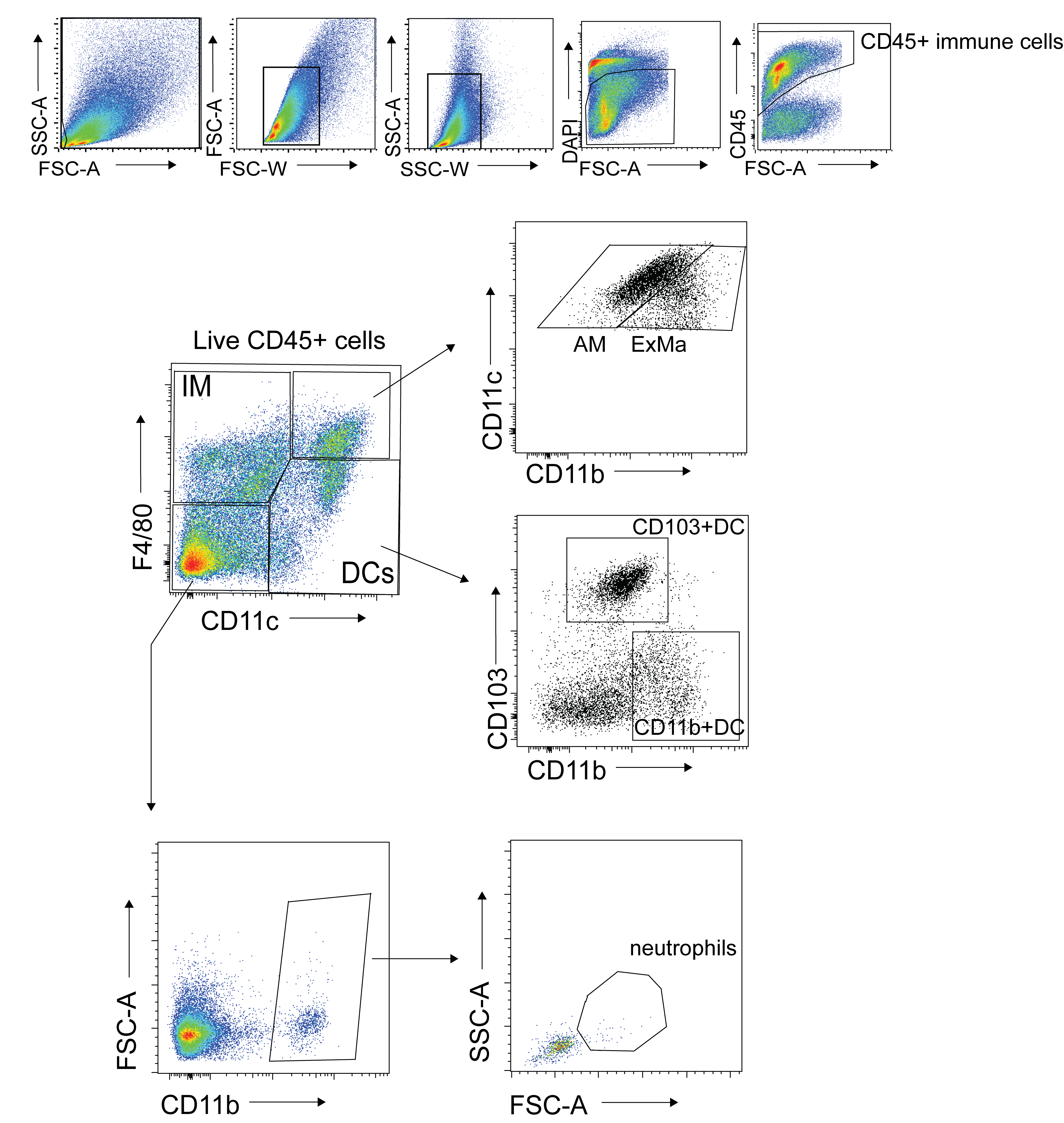

Supplement: Supplementary file 1 [file Image_1.tif]

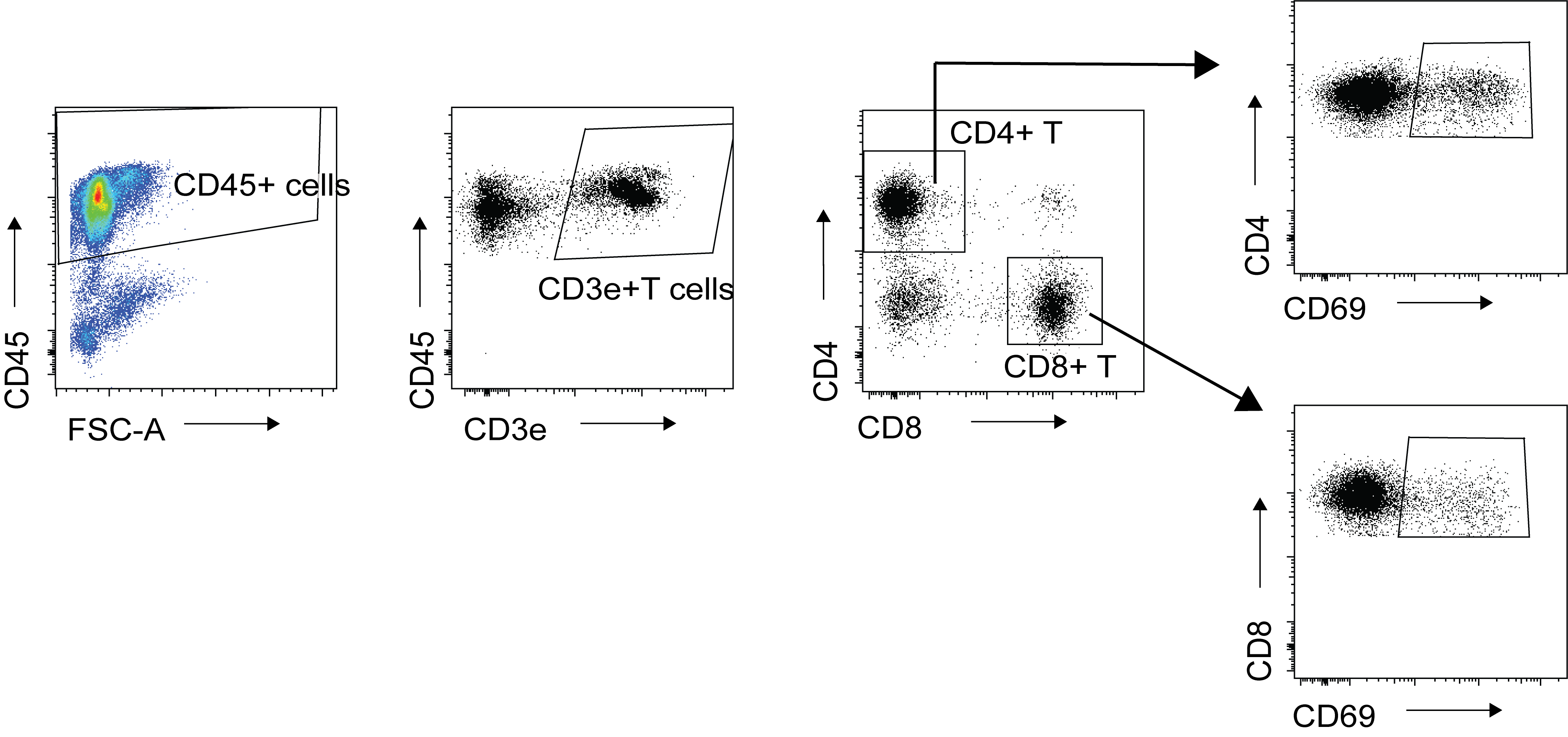

Supplement: Supplementary file 2 [file Image_2.tif]

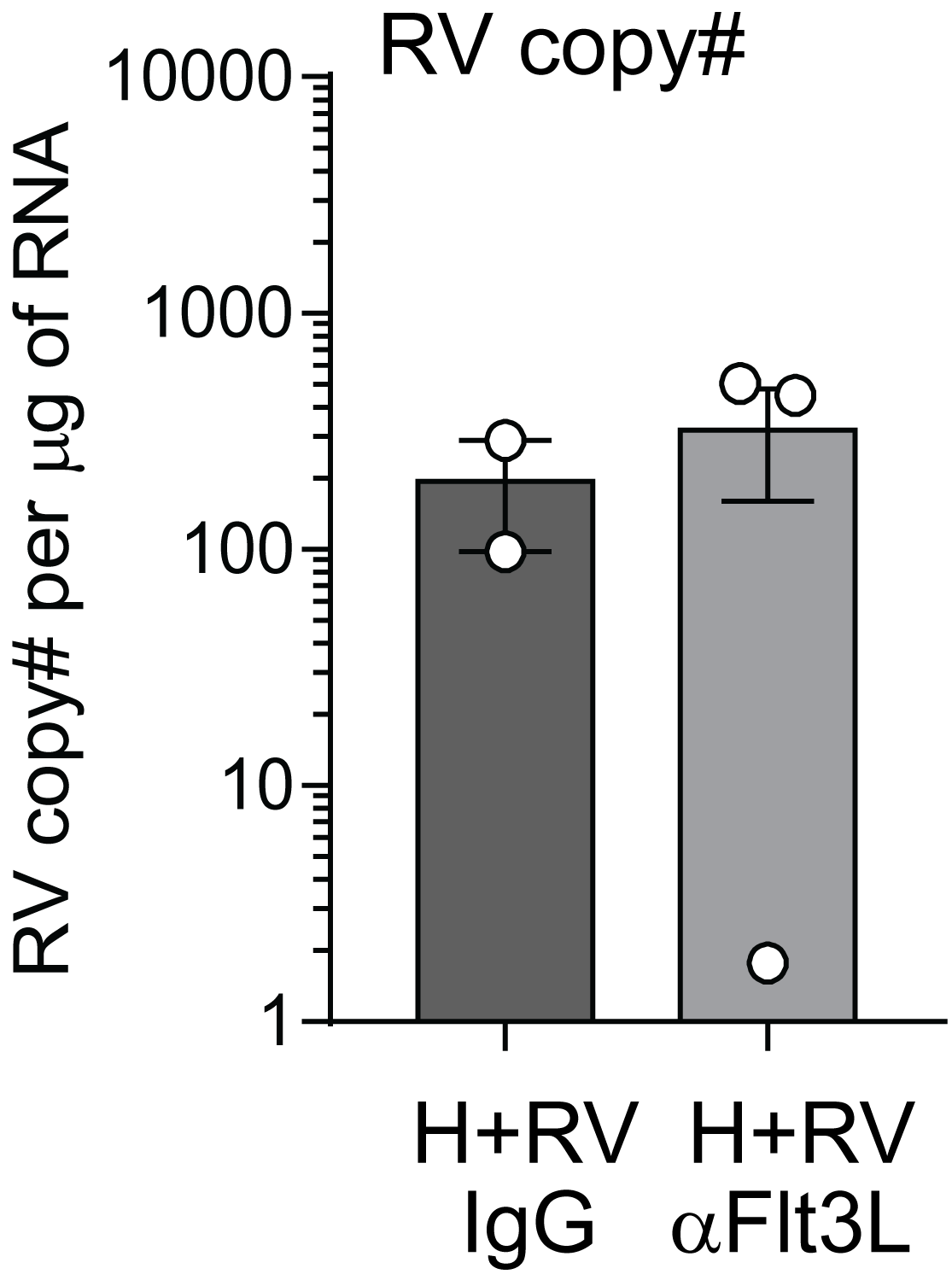

Supplement: Supplementary file 3 [file Image_3.tif]
